# Electrochemical and colorimetric sensing of P-xylene using doped C_60_ fullerenes: a dual approach to medical and environmental applications

**DOI:** 10.1038/s41598-025-30115-0

**Published:** 2025-12-03

**Authors:** Tareq Nafea Alharby, Muteb Alanazi, Jowaher Alanazi

**Affiliations:** 1 Department of Clinical Pharmacy, College of Pharmacy, University of Ha’il, Ha’il, 81442 Saudi Arabia; 2 Department of Pharmacology and Toxicology, College of Pharmacy, University of Ha’il, Ha’il, 81442 Saudi Arabia

**Keywords:** P-xylene detection, Prostate cancer biomarker, Electrochemical sensor, Environmental pollutant, DFT, Chemistry, Environmental sciences, Materials science

## Abstract

P-xylene is a type of aromatic hydrocarbon that has growing biomedical and environmental importance. It has been identified as a putative biomarker for prostate cancer and its fast and selective detection in biological fluids (especially urine and blood) is critical in the diagnosis and monitoring of the disease. Similarly, removal of p-xylene from industrial effluents and wastewater is an important environmental consideration due to its toxicity and persistence. These reasons emphasize the importance of developing and calculating the performance of efficient adsorbent and electrochemical sensors for p-xylene. Here, the adsorption and sensing performance of three fullerene-based nanostructures (C_60_, BC_59_, NC_59_) were computationally studied for p-xylene using electronic structure calculations, charge transport analysis, dipole moment calculations, and non-covalent interaction (NCI/RDG) maps. There were significant changes in electrical conductivity induced by adsorption and transduction was strongly analyte-dependent: C_60_ from 1.92 × 10^− 5^ to 2.93 × 10^− 4^ S/m (C_60_ @ p-xylene), BC_59_ from 1.18 × 10^− 2^ to 1.81 × 10^− 1^ S/m (BC_59_ @ p-xylene), NC_59_ from 3.68 × 10^− 3^ to 2.07 × 10^− 3^ S/m (NC_59_@ p-xylene). Recovery times were ultrafast for all three complexes with the fastest recovery time being for NC_59_ @ p-xylene (3.5 × 10^− 7^ s), C_60_ = 9.8 × 10^− 7^, and BC_59_@ p-xylene (1.9 × 10^− 5^ s). Dipole-moment analysis showed significant polarization upon adsorption for the doped systems. The dipole moment increased from 1.50 to 3.91 D in BC_59_ and from 1.39 to 3.05 D in NC_59_. NCI and RDG analyses found that C_60_@p-xylene is mainly affected by weak van der Waals forces. BC_59_@p-xylene shows stronger π-π interactions. NC_59_@p-xylene has intermediate but improved attractive interactions because of nitrogen doping. These trends are consistent with the adsorption energy ranking BC_59_ > C_60_ > NC_59_ and highlight the changes in sensor response and recovery behavior. This study shows that BC_59_ has the strongest adsorption and is suitable for environmental adsorption and removal of p-xylene. NC_59_, on the other hand, has extremely high conductivity modulation and ultrafast recovery, along with reasonable adsorption. This makes NC_59_ the most promising candidate for detecting p-xylene in early prostate cancer detection.

## Introduction

 The significance of volatile aromatic hydrocarbons (VAHs) cannot be overstated, as they are ubiquitous in industrial, environmental, and biological systems^1,2^. Compounds such as benzene, toluene, ethylbenzene, and xylenes are found in solvent products, petroleum products, and emissions from combustion processes^3,4^. Due to their volatility, they can disperse into the atmosphere quickly and cause adverse health effects through inhalation exposure, or by bioaccumulation within contaminated environments. Due to their slightly polar nature with hydrophobic properties, they can persist in biological systems, ultimately contributing to a number of adverse toxicological effects such as carcinogenicity, neurotoxicity, and consequences on respiratory health. For these reasons, VAHs are not only relevant for the monitoring of environmental contamination, but they also feature as important indicators used in biomedical diagnosis^5,6^. Para-xylene (p-xylene) is a noteworthy VAH due to its relevance in both the environmental and biomedical domains.

Jimenez-Pacheco et al. conducted an analysis of urinary volatile organic compounds (VOCs) collected post-prostate massage from patients with prostate cancer (PCa) and compared the profiles with those from individuals diagnosed with benign prostatic hyperplasia (BPH). Their results revealed a significant elevation of p-xylene in PCa urine samples (*p* = 0.002), along with increased levels of furan and phenol, suggesting a distinct metabolic signature associated with malignancy^7^. Also, in the field of environment, G Saggu et al. examined the effects of xylene and its derivatives as factors affecting the environment and human health^8^. Therefore, the accurate detection and identification of p-xylene are essential not only for environmental monitoring and public health protection but also for advancing diagnostic techniques in cancer research.

Given its dual relevance in environmental pollution and disease diagnostics, the development of efficient detection systems for p-xylene has become increasingly critical. Traditional analytical methods such as gas chromatography-mass spectrometry (GC-MS) offer high sensitivity and accuracy but are often limited by high costs, time-consuming procedures, and the need for sophisticated instrumentation and trained personnel^9,10^. In contrast, emerging sensor-based approaches (particularly electrochemical and colorimetric sensors) offer a promising alternative, providing rapid, cost-effective, and portable solutions for real-time monitoring^11^.

Beyond electrochemical and colorimetric sensors, researchers have looking toward other novel materials and methods for detecting volatile organic compounds, encompassing Surface Enhanced Raman Spectroscopy (SERS), Field-Effect Transistor (FET) sensors, and luminescence-based techniques^12^. Apart from offering their respective unique advantages for highly sensitive and selective detection (typically at low concentrations and/or in complex matrices), these methods have their own drawbacks (see Table 1)^13^.

**Table 1:** Comparison of different sensing techniques for analyte detection, highlighting their principles, advantages, and challenges.

Technique	Principle	Advantages	Challenges
SERS	Enhances Raman scattering via metallic nanostructures	Extremely sensitive, able to detect low concentrations	Requires precise fabrication of nanostructures, can be sensitive to environmental changes
FET	Measures electrical signal changes from interactions with analytes	Sensitivity and, acceptable response time, real-time monitoring	Requires well-designed interfaces, potential issues with stability
Luminescence-based	Uses light emission to detect molecules	Non-invasive, real-time monitoring, high selectivity	Often requires careful calibration, may have interference from background fluorescence

Therefore, it seems that the development of efficient sensors for accurate detection of paraxylene could facilitate early disease diagnosis and contribute to pollution control strategies.

Several studies have addressed the challenge of detecting p-xylene through the synthesis of electrochemical sensors. For instance, R. Guo et al. synthesized regular mesoporous ZnCr_2_O_4_, which demonstrated excellent sensing performance for highly sensitive and selective detection of p-xylene at parts-per-billion (ppb) levels. This material benefited from its high surface area and tailored pore structure, enabling effective adsorption and signal response to p-xylene vapor^14^. In a separate study, D. Yuan et al. introduced microporous biochar as a promising adsorptive material for the detection of gaseous p-xylene. The biochar sensor leveraged its high porosity and surface functionality to facilitate strong interactions with p-xylene molecules, thus offering an efficient and eco-friendly approach to vapor-phase sensing^15^. These advances underscore the ongoing effort to develop novel materials for the real-time monitoring of p-xylene in environmental and industrial settings.

Recently, there has been interest in using carbon nanostructures (specifically graphene, carbon nanotubes, and fullerenes) to build electrochemical and colorimetric sensors. This interest has been primarily due to their unique physicochemical properties, such as their substantial surface area, outstanding electrical conductivity, and adjustable surface chemistry^16–19^. Among the various fullerenes, the C_60_ fullerene has particularly stood out by virtue of its high electron affinity, symmetrical structure, and engagement in charge transfer interactions which provides advantages over other carbon-based materials in sensor applications. Numerous studies report that fullerene C60 functionalization enhances its electronics and adsorption capabilities. For instance, C. Nattagh et al. reported that C_60_ adsorption toward aspirin and other target molecules became more effective after C_60_ was boron (B) and nitrogen (N) doped^20^. Similarly, M.D. Esrafili et al. reported that B- and N-doped fullerenes C_60_ had superior adsorption of the toxic gases NO and NO2 as compared to un-doped C_60_, reinforcing the value of functionalization in expanding C60 applications^21^. These studies illustrate the importance of heteroatom doping to modify the electronic architecture of fullerenes for sensing.

Also, compared to experimental synthesis, computational chemistry provides a quicker, cheaper, and more accurate way to explore and improve nanomaterials. These computational methods, using density functional theory (DFT) and quantum theory of atoms in molecules (QTAIM), offer valuable insights into the electronic behavior, interaction mechanisms, and binding properties of sensor-analyte systems. This enables better design before laboratory synthesis^22–25^. Given the environmental and health importance of detecting p-xylene, along with the known sensing capabilities of pristine and doped C_60_ fullerenes, this study focuses on examining the interaction of p-xylene with C_60_ and its B- and N-doped derivatives. This research is a step toward creating a new class of highly sensitive sensors. To achieve this, DFT and QTAIM analyses were used to assess the electronic interactions and bonding properties involved. We believe that the findings from this research will lead to better and more selective p-xylene detection technologies based on modified carbon nanostructures.

## Computational details

**Fig. 1:**
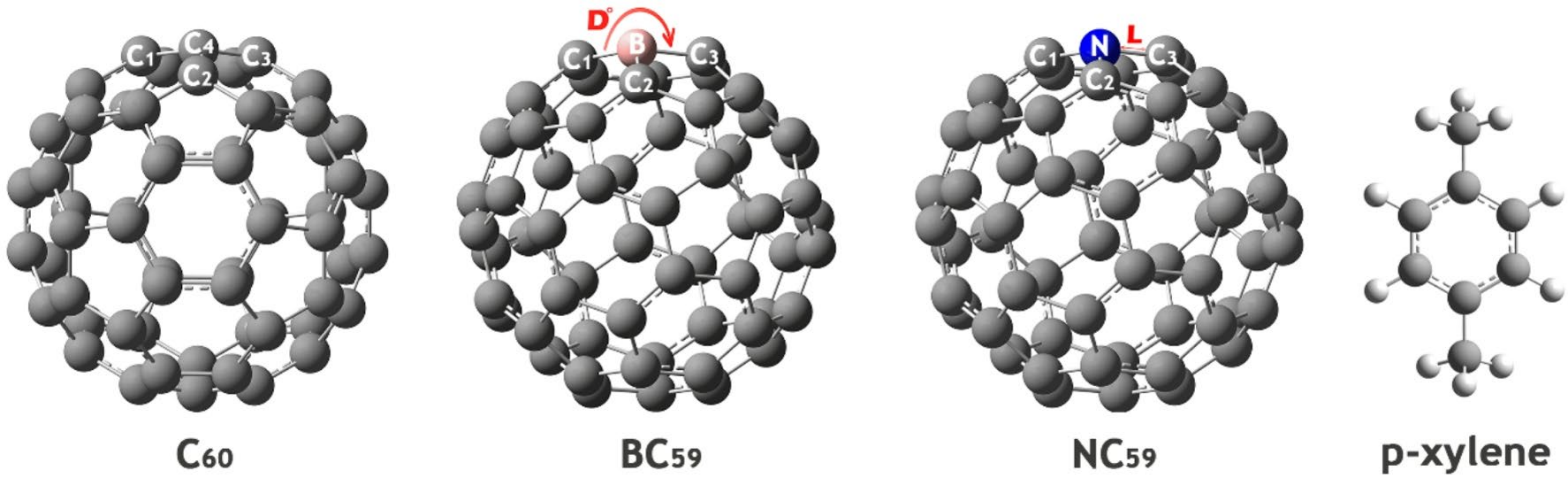
The optimized geometry of each of the structures studied in this work.

In this study, the molecular structures of para-xylene (p-xylene), pure C_60_ fullerene, and its doped derivatives (boron-doped (BC_59_) and nitrogen-doped (NC_59_) fullerenes) were initially designed using GaussView 6.0 software (See Fig. 1). Then, all geometries were fully optimized using Gaussian 09 W software (Fig. 1)^26,27^.

Geometry optimizations were performed using Density Functional Theory (DFT) with the B97D functional and the 6-31G* basis set^28,29^. To match the computational conditions with the planned biomedical application for detecting p-xylene in biological fluids, the water phase was chosen using the Conductor-like Polarizable Continuum Model (CPCM). This model approximates the polar environment of urine and blood, where p-xylene is seen as a potential biomarker for prostate cancer. Using an implicit water model allows the calculated adsorption energies, electronic properties, and sensor-analyte interaction features to better represent system behavior under conditions relevant to physiology. This ensures that the predicted sensing performance of both pristine and doped C60 structures reflects the aqueous environments in which real diagnostic tests would occur^30,31^. The B97D functional was specifically selected because it includes empirical dispersion corrections, which are necessary for accurately describing van der Waals and non-covalent interactions that commonly occur in fullerene-based systems^32,33^. Notably, the B97D functional has been previously validated for fullerene C_60_, showing results that strongly match available experimental data, particularly regarding geometry and electronic properties^34^. This makes it a trustworthy and suitable choice for this study.

To confirm the stability of the optimized structures, frequency calculations were done at the same theoretical level (B97D/6-31G*, CPCM/water). The absence of imaginary frequencies in all cases indicates that the structures are real minima on the potential energy surface. This confirms their thermodynamic stability^35,36^.

The stability of the designed systems was evaluated through cohesive energy calculations, as defined in Eq. (1).

1 $$\:E_{{Coh}} = - (E_{{tot}} - \sum {\:_{i} } n_{i} E_{i} )/n$$

E_tot_: Total electronic energy of the optimized molecule or complex. ∑_𝑖_𝑛_𝑖_𝐸_𝑖_: The summation of the total energies of the isolated atoms making up the structure. Here, 𝐸𝑖 is the energy of the atom, and 𝑛𝑖 is the number of atoms of that type in the structure. 𝑛: Total number of atoms in the structure^37,38^.

The electronic and quantum properties of the designed structures were calculated using key descriptors, including energy gap (HLG), chemical softness (S), chemical hardness (η), chemical potential (µ), maximum charge transfer value (ΔNmax), and electrophilicity-based charge transfer (ECT), according to Eq. 2 to 7, respectively^39–45^.

2 $$\:HLG = \left| {E_{{HOMO}} - E_{{LUMO}} } \right|$$

3 $$\:\eta = ( - E_{{HOMO}} - ( - E_{{LUMO}} \:))/\: - 2$$

4 $$\mu = - ( - E_{{HOMO}} + ( - E_{{LUMO}} ))/2$$

5 $$S = 1/2\eta$$

6 $$\Delta N_{{\max }} = - \mu /\eta \:$$

7 $$ECT = \left( {\Delta \:N_{{\max }} } \right)_{{\alpha \:}} - \left( {\Delta \:N_{{\max }} } \right)_{{\beta \:}}$$

E_HOMO_ and E_LUMO_ represent the energies of the highest occupied (HOMO) and lowest unoccupied (LUMO) molecular orbitals, respectively, and are key indicators of a molecule’s electron-donating and -accepting abilities.

ΔN_max_ represents the maximum charge transfer, with α for the complex and β for the (B/N)-doped C_60_. A positive ECT means the sensor donates electrons to furan, while a negative ECT indicates electron transfer from p-xylene to the sensor^44,46^.

The sensing mechanism of each designed structure was calculated and evaluated using key parameters including adsorption energy (Eads), recovery time (τ), and electrical conductivity (σ) (Eqs. 8, 9, 10)^47–49^.

8 $$\:E_{{ads}} = E_{{\left( {R - } \right)C20@p - xylene}} - \left( {E_{{p - xylene}} + E_{{\left( {R - } \right)C60}} } \right) + E_{{BSSE}}$$

9 $$\tau \: = V_{0}^{{ - 1}} \times \:\exp ( - \frac{{E_{{ads}} }}{{k_{B} T}})$$

10 $$\:\sigma \: = AT^{{3/2}} e^{{( - HLG/2KT)}}$$

In Eq. (8), the adsorption energy (Eads) is computed using the total energy of the p-xylene–sensor complex (E_(R−)C60@p−xylene_), the energies of the isolated p-xylene (E_p−xylene_) and the sensor (E_(R−)C60_), along with the basis set superposition error correction (E_BSSE_). Equation (9) defines the recovery time (τ) based on the adsorption energy (Eads), where V_0_ is the attempt frequency (typically ~ 10^12^ s^− 1^), kB is the Boltzmann constant (8.617 × 10^− 5^ eV/K), and T is the temperature (commonly 298 K). Lastly, Eq. (10) describes the electrical conductivity (σ), where A is the Richardson constant (6 × 10^5^ A.m^− 2^.K^− 2^), and T is the temperature (298 K)^50,51^.

These calculations were systematically performed to investigate the adsorption behaviour, electronic properties, and sensing potential of C60 fullerene and its functionalized derivatives for the detection of paraxylene, with the aim of contributing to the development of diagnostic tools for environmental protection and advances in diagnostic methods for diseases such as prostate cancer.

## Results and discussion

### Bond lengths and bond angles

Examining bond lengths and bond angles in doped structures is crucial, as doping alters the electronic distribution, particularly the displacement of π electrons, which directly affects the molecular geometry. Changes in bond length and angle reflect how the dopant perturbs the delocalized π-electron system, influencing the structural stability, reactivity, and overall electronic properties of the material. These geometric variations can significantly impact the sensor’s interaction with target molecules, thereby affecting its sensitivity and selectivity^52,53^. Therefore, the bond lengths and bond angles for each designed structure are reported in Table 2.

**Table 2:** Bond length (A) and bond angle (Dᵒ) of some important atoms in each of the studied structures.

Structure	Bond lengths (Å)		Bond angles (°)	
C_60_	C1-C4	1.45	C1-C4-C2	119.98
	C2-C4	1.40	C1-C4-C3	107.99
	C3-C4	1.45	C2-C4-C3	119.99
BC_59_	C1-B	1.53	C1-B-C2	118.46
	C2-B	1.55	C1-B-C3	118.46
	C3-B	1.55	C2-B-C3	106.26
NC_59_	C1-N	1.40	C1-N-C2	119.22
	C2-N	1.42	C1-N-C3	119.22
	C3-N	1.42	C2-N-C3	107.411

The bond length and bond angle data presented in Table 1 reveal significant structural changes in the fullerene framework upon doping with boron and nitrogen atoms. In the pristine C_60_ structure, the C-C bond lengths range from 1.40 to 1.45 Å, and the corresponding bond angles are close to the ideal sp^2^ hybridization angles, ranging from approximately 108° to 120°, reflecting the symmetrical and delocalized π-electron network characteristic of fullerene.

Upon boron doping (BC_59_), the B-C bond lengths increase to 1.53–1.55 Å, indicating a weakening or elongation of the bonds due to the introduction of a boron atom, which has fewer valence electrons than carbon. This electron deficiency disrupts the π-electron distribution, leading to local geometric distortion. The bond angles around the boron center shift slightly from the pristine values, with angles such as C1-B-C2 and C1-B-C3 decreasing to 118.46°, and C2-B-C3 narrowing further to 106.26°, reflecting a localized strain and rearrangement in the bonding environment.

In contrast, nitrogen doping (NC_59_) results in shorter N-C bonds (1.40–1.42 Å), which can be attributed to nitrogen’s higher electronegativity and its ability to attract π-electrons. This leads to a more compact bonding region compared to the B-doped case. The bond angles around nitrogen (119.22° for C1-N-C2 and C1-N-C3, and 107.41° for C2-N-C3) remain relatively close to those of pristine C_60_, suggesting less geometric distortion than in the BC_59_ structure.

These variations confirm that doping significantly affects the geometry of fullerene by displacing π-electrons, which alters the bond lengths and angles.

### Cohesive energy

**Fig. 2:**
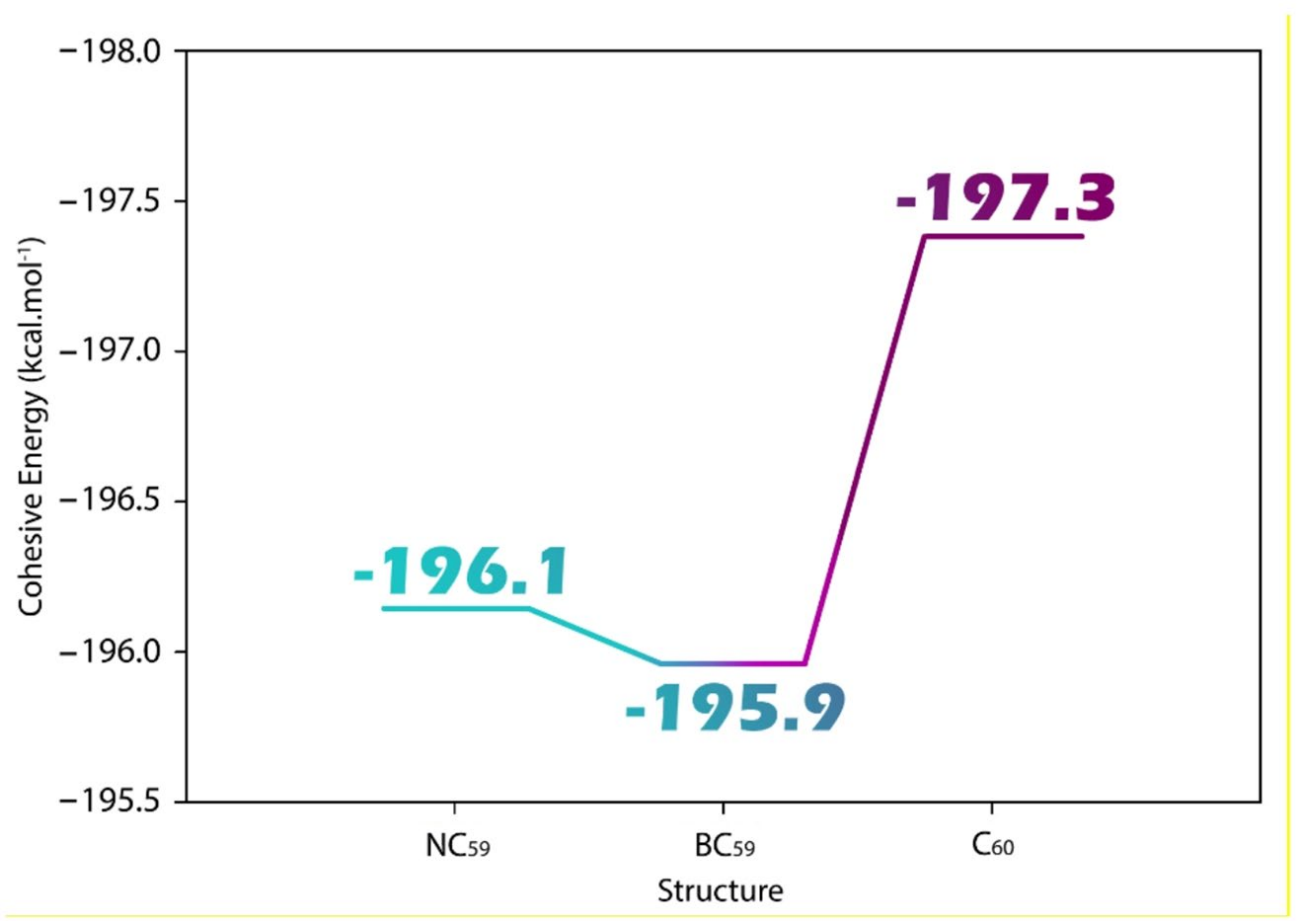
Effect of doping on coherence energy in C_60_ fullerene and its forms of doping with boron and nitrogen (BC_59_ and NC_59_).

Cohesive energy is defined as the amount of energy required to disassemble a compound into its individual atoms, representing the overall stability of a structure. Examining cohesive energy after doping is essential because it provides insight into how the introduction of dopant atoms, such as boron or nitrogen, affects the structural integrity and stability of the material. A lower or more negative cohesive energy generally indicates a more stable configuration^54–56^. Figure 2 illustrates the changes in cohesive energy observed after doping in each of the designed structures, highlighting the impact of dopants on structural stability.

Based on the cohesive energy values reported in Fig. 2, it is evident that doping slightly reduces the structural stability of the pristine C_60_. The cohesive energy of C_60_ is calculated as −197.3 kcal/mol, indicating a highly stable configuration. Upon doping with boron (BC_59_), the cohesive energy decreases to −195.9 kcal/mol, while nitrogen doping (NC_59_) results in a slightly higher cohesive energy of −196.1 kcal/mol. This reduction in cohesive energy for both doped structures suggest a minor destabilization due to the introduction of heteroatoms, which disrupts the original π-electron delocalization and bonding network of the fullerene cage. However, the difference is not substantial, indicating that the doped structures remain thermodynamically stable. The slightly higher cohesive energy in NC_59_ compared to BC_59_ also suggests that nitrogen doping is marginally more favorable in terms of maintaining structural integrity.

### Molecular electrostatic potential (MEP) analysis

**Fig. 3:**
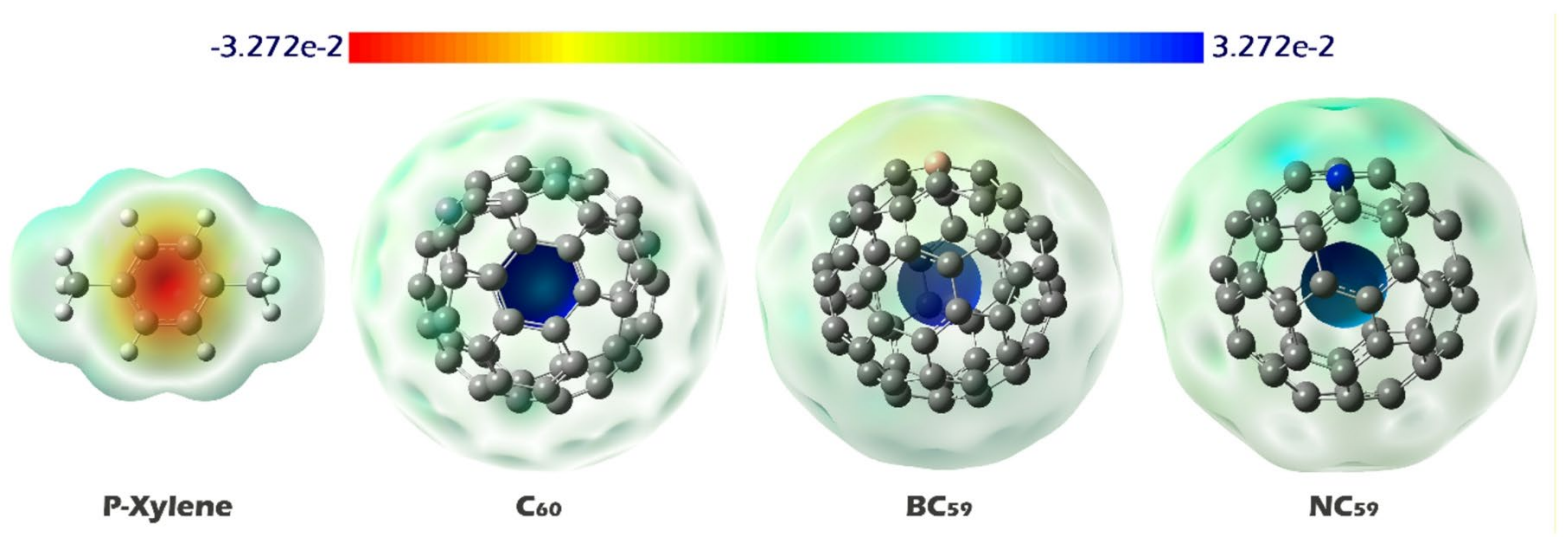
MEP contours for p-xylene, C_60_ fullerene, and its doped forms (BC_59_ and NC_59_).

Molecular Electrostatic Potential (MEP) contours are important for identifying potential interaction sites between molecules. This is especially true in adsorption and sensing studies. These contours show the distribution of electrostatic potential across the molecular surface using color gradients. Red areas indicate high electron density, or negative potential, while blue areas represent zones that lack electrons, or positive potential. By looking at these colored regions, one can predict where electrophilic or nucleophilic attacks may occur and spot possible interaction points with analyte molecules. This helps clarify the nature of intermolecular forces^57,58^. Therefore, MEP contours were plotted to identify the most likely interaction points for designing sensor-analyte complexes in each of the structures shown in Fig. 3.

**Fig. 4:**
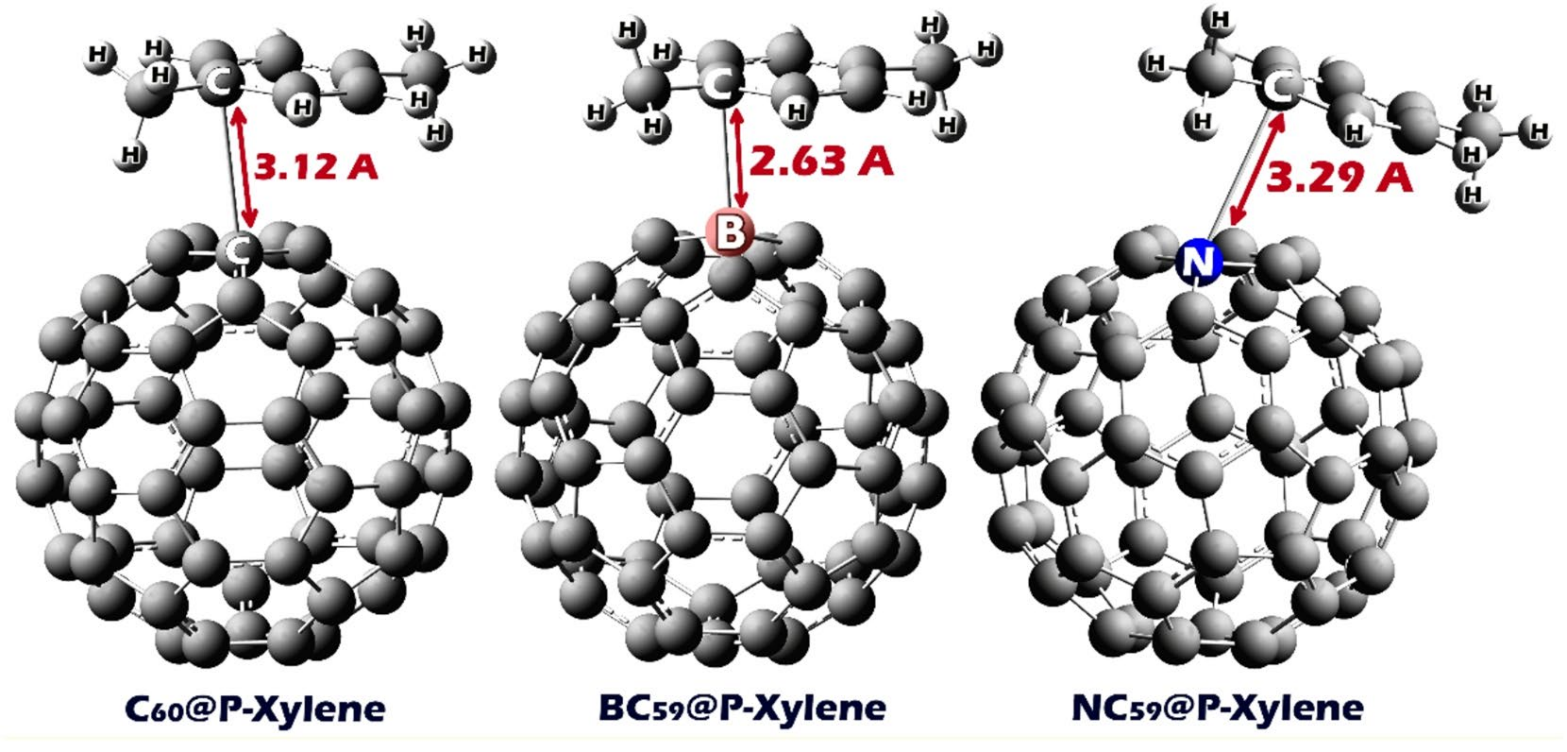
Optimized geometry of each of the complexes designed in this work.

As shown in the molecular electrostatic potential (MEP) maps, the interaction of p-xylene with fullerenes, especially primary C_60_ and its doped derivatives (BC_59_ and NC_59_), is expected to occur mainly in regions with positive electrostatic potential. In particular, p-xylene, with its electron-rich aromatic ring, interacts favorably with these electrophilic sites. The MEP analysis showed that the pristine C_60_ exhibits a strong blue region indicative of an electrophilic site. This behavior aligns with studies that have highlighted the significance of such interactions between electron-rich molecules (like aromatic hydrocarbons) and electron-deficient regions on carbon-based nanomaterials. Upon boron doping (BC_59_), the positive potential becomes slightly less intense, but the interaction potential remains strong near the boron atom. On the other hand, nitrogen doping (NC_59_) reduces the overall positive electrostatic potential, but still allows significant interaction near the nitrogen atom due to its electronegativity. These findings are consistent with the literature, which suggests that functionalization or doping of fullerenes can modulate the electrostatic potential, thereby influencing the adsorption characteristics with target molecules^59^. Based on these findings, the desired complexes were designed and their geometric structure was optimized (see Fig. 4).

Figure 4 illustrates the optimized geometries and interaction distances between p-xylene and the fullerene-based structures (C_60_, BC_59_, and NC_59_). The bond length between the p-xylene molecule and the pristine C_60_ surface has been measured at 3.12 angstroms, indicating a moderate adsorption driven mainly by van der Waals forces. Upon boron doping (BC_59_@p-Xylene), the interaction distance significantly decreases to 2.63 Å, indicating a stronger interaction. This reduction in bond length is attributed to the electron-deficient nature of boron, which enhances the electrophilic character of the fullerene surface and promotes stronger adsorption of the electron-rich p-xylene. In contrast, nitrogen doping (NC_59_@p-xylene) leads to an increase in bond length to 3.29 Å, the longest among the three complexes. This elongation reflects a weaker interaction, likely due to the electron-rich nature of nitrogen, which repels the π-electron cloud of p-xylene and reduces the overall adsorption strength.

### Reactivity parameters

The consideration reactivity indicators are essential for the development of electrochemical sensors because they all influence the sensitivity and function of the electrochemical sensor. The energy gap (HLG), indicates the reactivity and electronic conductivity of the materials (smaller energy gaps are typically higher sensitivity)^60^. Chemical softness (S) and chemical hardness (η) indicate the system’s ability to donate or accept electrons, generally softer systems are more reactive and substitutable in charge transfer process. The chemical potential (µ) indicates the inclination of electrons to escape from the chemical system which is related to the strength and selectivity of the interaction with the analytes^61^. Maximum charge transfer (ΔNmax) indicates the maximum ability of the sensor to donate or accept charge, while electrophilicity-based charge transfer (ECT) measures the direction of charge transfer for the electrons in chemical reaction process for the sensor-analyte^62^. These reactivity indicators are related and provide a measure of the expected reactivity, selectivity, and efficiency of the sensor.

**Table 3:** Calculated electronic reactivity descriptors of C60, BC59, NC59, and their p-xylene complexes.

Structure	LUMO	HOMO	HLG	η	µ	S	$\:{\varDelta\:\varvec{N}}_{\varvec{m}\varvec{a}\varvec{x}}$	ECT
C_60_	−3.7	−5.38	1.68	0.84	−4.54	0.59	5.40	-
BC_59_	−3.58	−4.93	1.35	0.67	−4.25	0.74	6.30	-
NC_59_	−3.79	−5.2	1.41	0.70	−4.49	0.70	6.37	-
C_60_@ P-Xylene	−3.54	−5.08	1.54	0.77	−4.31	0.64	5.59	−0.19
BC_59_@ P-Xylene	−3.47	−4.68	1.21	0.60	−4.07	0.82	6.73	−0.43
NC_59_@ P-Xylene	−3.35	−4.08	0.73	0.36	−3.71	1.36	10.17	−3.80

**Fig. 5:**
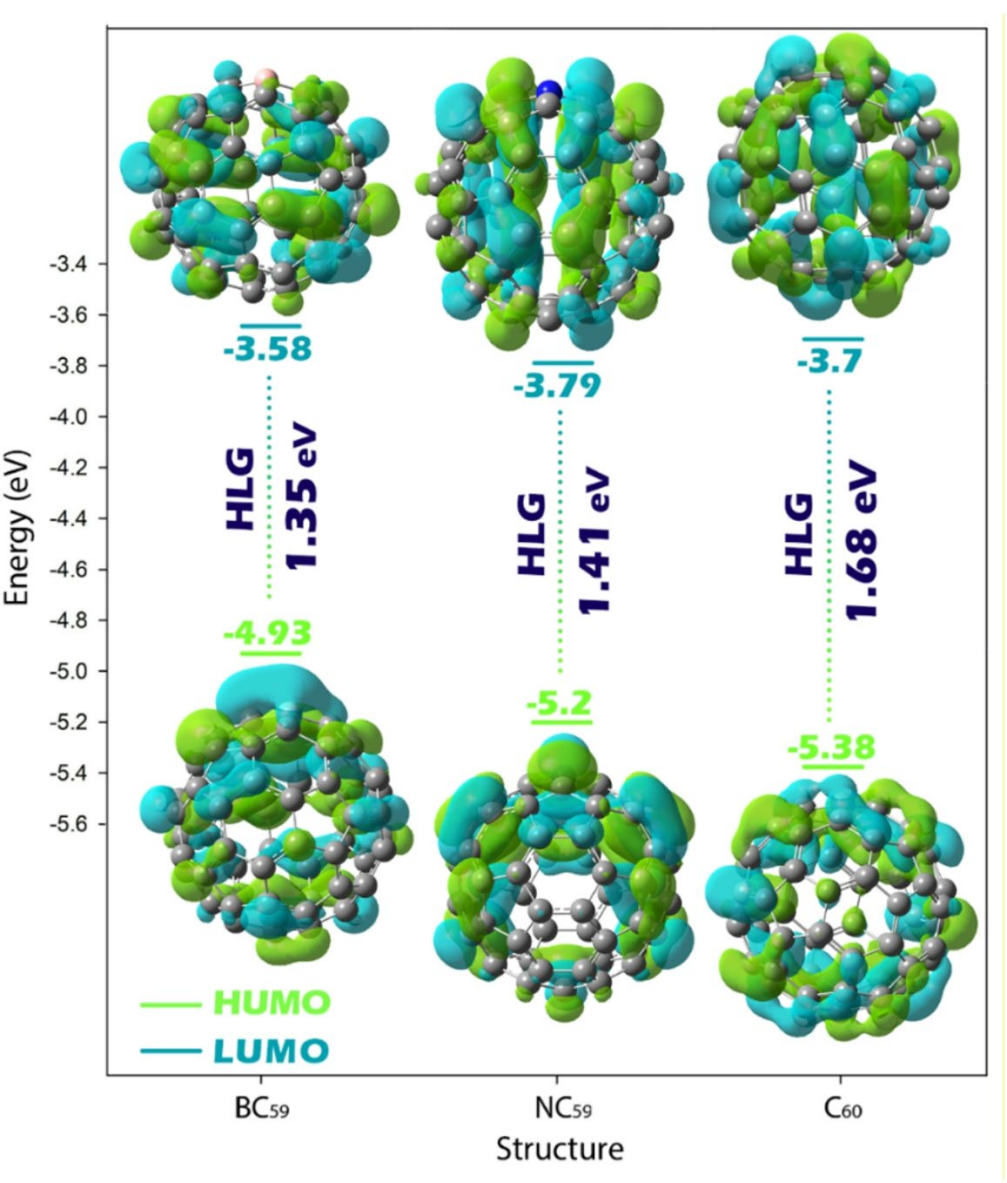
The trend of energy gap changes for pristine C_60_ and its doping forms, as well as the distribution of HOMO and LUMO orbitals.

The information in Table 3 provides a useful comparison of the electronic and reactivity descriptors for fullerenes that are either pristine (C_60_) or doped (BC_59_ and NC_59_), and when they are complexed with p-xylene. The HOMO and LUMO energy levels show that pristine C_60_ had a HOMO of −5.38 eV and LUMO of −3.70 eV resulting in an energy gap (HLG) of 1.68 eV. The energy gap calculated for C_60_ in this study (1.68 eV) shows strong consistency with the value reported by Rabenau et al. (1.86 eV) based on temperature-dependent microwave conductivity measurements. This close agreement supports the accuracy of the function employed in our calculations. It confirms the reliability of the computational methodology applied to the remaining fullerene-based structures (BC_59_ and NC_59_) examined in this work^63^. Upon doping, the HOMO level increases (i.e., becomes less negative) in BC_59_ (−4.93 eV) and NC_60_ (−5.20 eV), while the LUMO changes slightly to −3.58 eV (BC_59_) and − 3.79 eV (NC_59_). As a result, the HLG decreases to 1.35 eV in BC_59_ and 1.41 eV in NC_60_, indicating enhanced electrical conductivity compared to pristine C_60_ (see Fig. 5).

When interacting with p-xylene, all complexes show further narrowing of the energy gap, particularly in NC_59_@p-xylene, where the HLG drops sharply to 0.73 eV, suggesting significantly improved charge transport properties. BC_59_@p-xylene and C_60_@p-xylene also show reduced gaps of 1.21 eV and 1.54 eV, respectively, confirming enhanced reactivity and potential sensor activity upon interaction.

**Fig. 6:**
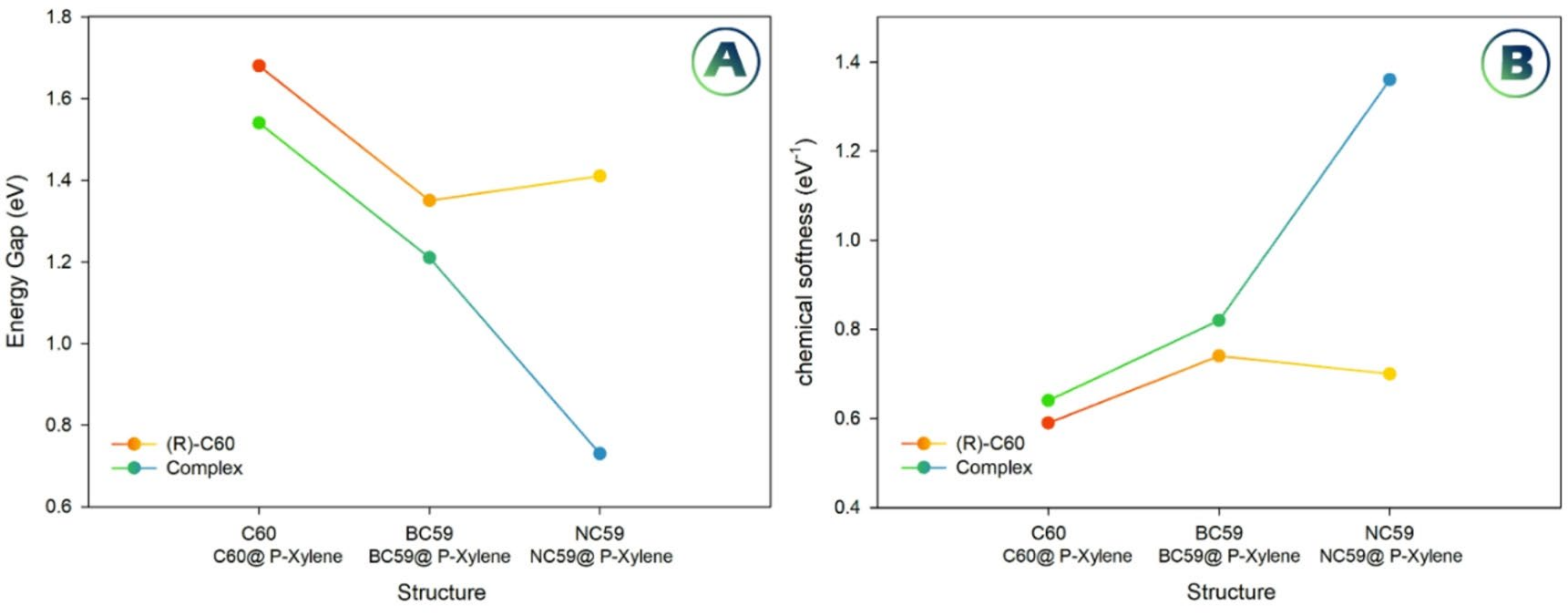
Changes in the energy gap (**A**) and chemical softness (**B**) in the presence and absence of p-xylene.

Chemical hardness (η) decreases after doping: from 0.84 eV in C_60_ to 0.67 eV in BC_59_ and 0.70 eV in NC_59_. After adsorption, η further reduces, especially in NC_59_@p-xylene (0.36 eV), suggesting that the complex becomes more reactive and more prone to electronic perturbation. Chemical softness (S), which is inversely related to hardness, shows the opposite trend, increasing from 0.59 (C_60_) to 0.74 (BC_59_) and 0.70 (NC_59_), and further rising to 1.36 in NC₅₉@p-xylene, confirming its enhanced polarizability and potential for sensing (Fig. 6).

Chemical potential (µ) becomes less negative after doping and adsorption, shifting from − 4.54 eV in C₆₀ to −4.25 eV (BC_59_) and − 4.49 eV (NC_59_), and further to −4.07 eV (BC_59_@p-xylene), −4.31 eV (C_60_@p-xylene), and − 3.71 eV (NC_59_@p-xylene). This upward shift indicates an increased tendency of the complexes to donate electrons, particularly in NC_59_@p-xylene (Fig. 6).

The maximum amount of charge transfer (ΔNmax) increases notably after doping and especially after interaction with p-xylene. It rises from 5.40 in pristine C₆₀ to 6.30 (BC_59_) and 6.37 (NC_59_), and further to 5.59 (C_59_@p-xylene), 6.73 (BC_59_@p-xylene), and 10.17 in NC_59_@p-xylene, showing that NC_59_ has the greatest capacity for charge transfer after interaction, a favorable feature for sensing applications.

Finally, electrophilicity-based charge transfer (ECT) for all complexes shows negative values, confirming charge transfer from p-xylene to the sensor. The ECT values are − 0.19 (C_60_@p-xylene), −0.43 (BC_59_@p-xylene), and − 3.80 (NC_59_@p-xylene). The significantly more negative ECT in NC_59_@p-xylene indicates the highest electron donation toward the sensor, confirming strong charge transfer and higher sensing potential of NC_59_.

Finally, although NC_59_ exhibits the most favorable reactivity profile (characterized by the lowest energy gap, highest chemical softness, and greatest charge transfer capacity), making it the most promising candidate for sensitive and efficient electrochemical sensing, BC_59_ also demonstrates remarkable electronic properties. The relatively low energy gap, high charge acceptance ability, and balanced electrochemical parameters of BC5_59_ confirm the strong sensing potential of this structure. Therefore, while NC_59_ shows excellent reactivity, a definitive conclusion regarding the most efficient sensor requires further analysis, which is discussed in the following sections.

**Fig. 7:**
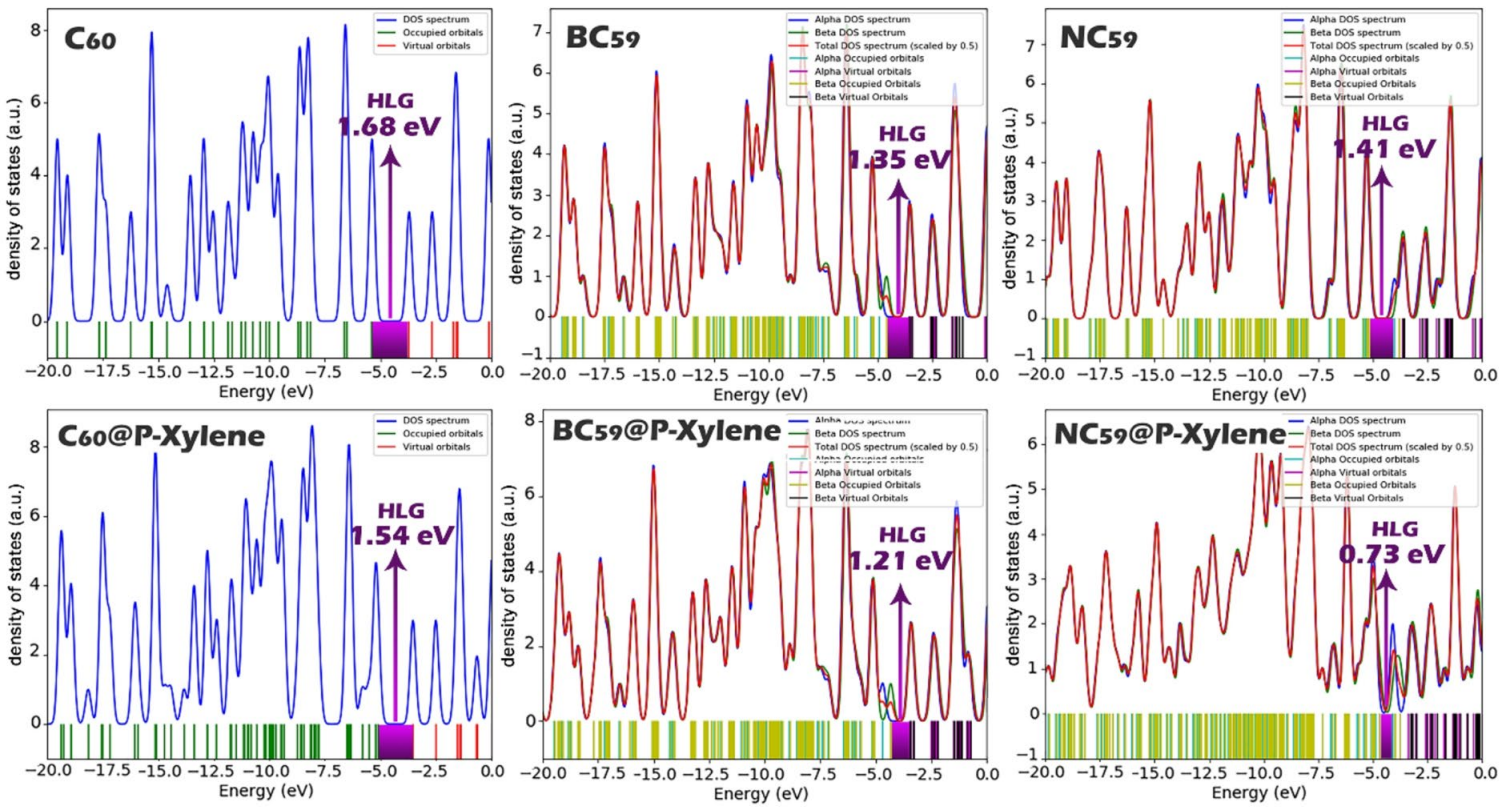
DOS plot for each of the structures designed in this work.

Density of States (DOS) graphs are useful graphics that show the area of the electronic states at various energy levels in a molecule, namely the location of the HOMO and LUMO and the gap between them^63–65^. In the present study, DOS graphs were made for each of the structures considered (pristine & doped fullerenes, and their p-xylene complexes) (Fig. 7). The DOS graphs show the energy levels and confirm the energy gaps (HLG) values presented in Table 2. The projection from the DOS images and the numerical values aligns and add credibility to the electronic properties computed, substantiating trends in reactivity and sensing behavior.

**Fig. 8:**
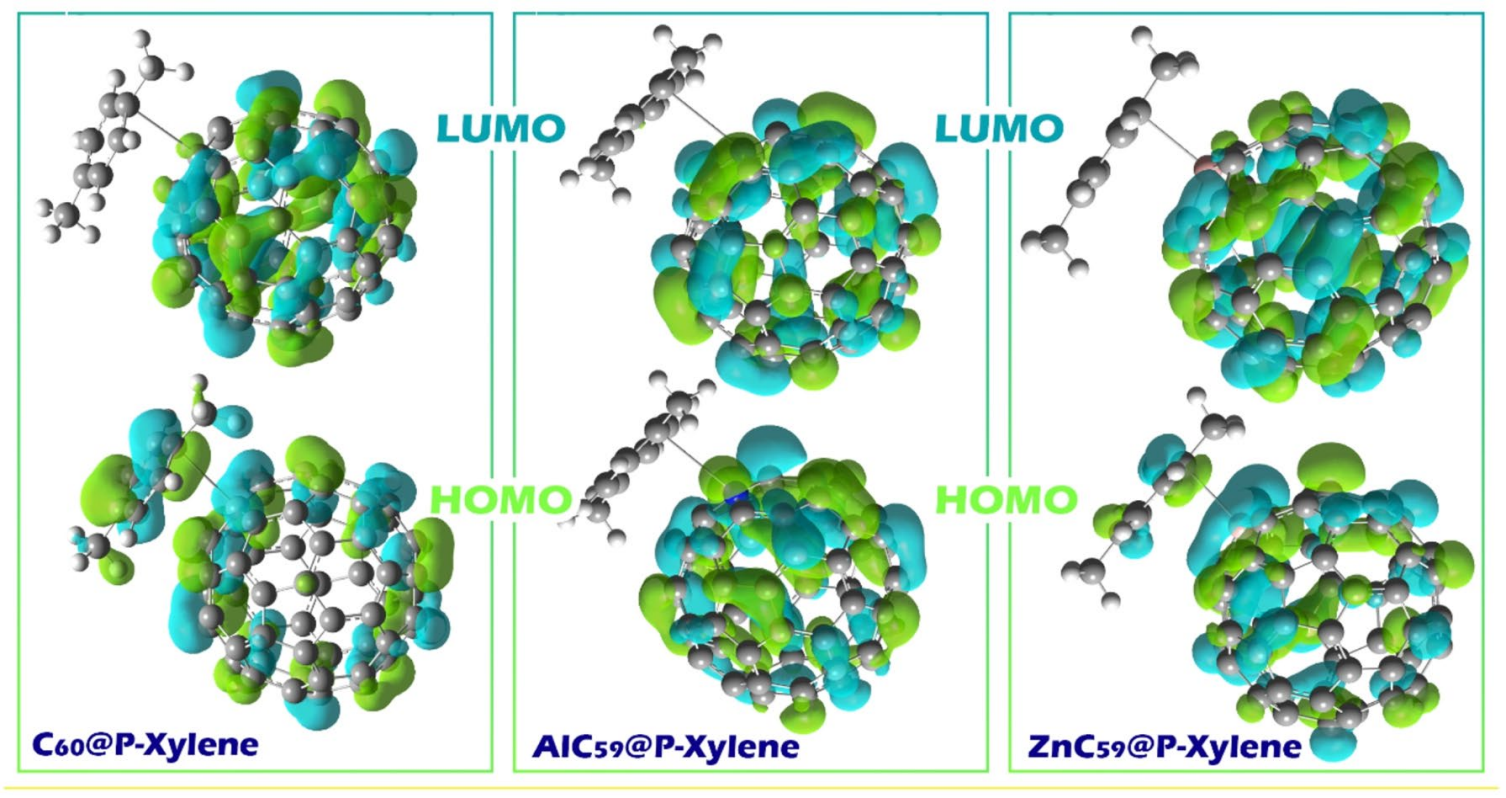
How HOMO and LUMO orbitals are distributed in each of the studied complexes.

The distribution of HOMO and LUMO orbitals in molecular complexes is critical for understanding the charge transfer behavior and electronic interactions between the sensor and the analyte. The spatial localization of these orbitals reveals the direction and likelihood of electron flow during adsorption, which directly influences the sensor’s sensitivity and efficiency (Fig. 8)^66,67^.

In the C_60_@p-xylene complex, the HOMO orbitals are delocalized across the entire complex, while the LUMO orbitals are primarily localized on the C_60_. This suggests that upon interaction, electrons from p-xylene (donor) can transfer into the LUMO of C_60_, indicating a donor–acceptor interaction and favorable charge transfer toward the sensor. In the NC_59_@p-xylene complex, both the HOMO and LUMO orbitals are localized on the fullerene, indicating that the electronic interaction is mostly confined to the sensor. This distribution implies a weaker charge transfer interaction with p-xylene, as the molecular orbitals responsible for electron transitions are not extended toward the analyte. This finding is consistent with the longer bond length and weaker adsorption energy observed for this complex. In contrast, in the BC_59_@p-xylene complex, the HOMO and LUMO orbitals are delocalized across the complex, with the LUMO specifically concentrated on the BC_59_ region. This orbital distribution suggests stronger electronic coupling and efficient charge transfer from p-xylene to the boron-doped sensor, supporting enhanced sensing behavior. The localized LUMO on BC_59_ further indicates that boron doping enhances the electron-accepting ability of the fullerene.

These orbital distribution results are consistent with the electrophilicity-based charge transfer (ECT) values reported in Table 2. Overall, analysis of these results shows that BC_59_ offers the most favorable orbital overlap for charge transfer, while NC_59_ exhibits limited interaction and C_60_ exhibits intermediate behavior.

### Sensor properties investigation

#### Adsorption energy, recovery time, and electrical conductivity

Adsorption energy (Eads), recovery time (τ), and electrical conductivity (σ) are some of the most critical factors of determining a structure’s performance as a chemical sensor. Adsorption energy refers to the strength and stability of the interaction between the sensor and analyte, while recovery time indicates how quickly the sensor can return to its original state after detection. Electrical conductivity determines the sensor’s ability to create a signal that can be measured electrically once the analyte has bound^68–71^. The mentioned parameters determine the sensitivity, reusability, and practicality of the sensor [Table 4].

**Table 4:** Electrical conductivity (𝝈), adsorption energy (Eads), basis set superposition error correction (EBSSE), and recovery time (𝝉) values in each of the studied structures.

Structure	Eads (kJ/mol)	E_BSSE_ (kJ/mol)	𝝉 (s)	(𝝈) (S/m)
C_60_	-	-	-	1.92 × 10^− 5^
BC_59_	-	-	-	1.18 × 10^− 2^
NC_59_	-	-	-	3.68 × 10^− 3^
C_60_@p-xylene	−34.30	12.71	9.8 × 10^− 7^	2.93 × 10^− 4^
BC_59_@p-xylene	−41.68	13.68	1.9 × 10^− 5^	1.81 × 10^− 1^
NC_59_@p-xylene	−31.71	12.92	3.5 × 10^− 7^	2.07 × 10^3^

The interaction of p-xylene with each of the designed sensors (C_60_, BC_59_, and NC_59_) yields a clear and consistent pattern in terms of adsorption capacity, charge transfer response, and desorption kinetics. Adsorption energies (kJ/mol) follow BC_59_@p-xylene (−41.68) < C_60_@p-xylene (−34.30) < NC_59_@p-xylene (−31.71), i.e., BC_59_ binds p-xylene most strongly, then C_60_, with NC_59_ the weakest binder. All three magnitudes fall in the moderate, largely physisorption regime typical for π-π/dispersion-dominated interactions, which is desirable for reversible capture/release cycles in sensing and for regenerable adsorbents in environmental applications. The electrical conductivity (σ, S/m) increases upon adsorption for all sensor, but the magnitude of the change is highly analyte-dependent (p-xylene). C_60_ rises from 1.92 × 10^− 5^ to 2.93 × 10^− 4^ S/m; BC_59_ from 1.18 × 10^− 2^ to 1.81 × 10^− 1^ S/m; and NC59 from 3.68 × 10^− 3^ to 2.07 × 10^3^ S/m. This indicates that while adsorption strengths rank BC59 > C_60_ > NC59, the transduction efficiency (how strongly the adsorption event perturbs charge transport) is overwhelmingly largest for NC_59_. Recovery times (τ, s) are ultrafast for all three complexes, with NC_59_@p-xylene the fastest (3.5 × 10^− 7^ s), followed by C_60_@p-xylene (9.8 × 10^− 7^ s), and BC_59_@p-xylene slower at 1.9 × 10^− 5^ s. The inverse trend between adsorption strength and recovery speed (stronger binding → longer τ) is physically sensible: BC_59_’s stronger adsorption slows desorption (still microseconds), whereas NC59’s weaker adsorption facilitates rapid recovery.

Finally, BC_59_ maximizes adsorption strength and, alongside C_60_, is best suited for environmental capture and remediation of p-xylene; NC_60_, despite its weaker binding, delivers a uniquely massive conductivity response and the fastest recovery, making it the preferred electrochemical sensing platform for p-xylene in urine and a strong candidate for early-stage prostate-cancer detection workflows contingent on appropriate clinical validation.

#### Dipole moment

**Fig. 9:**
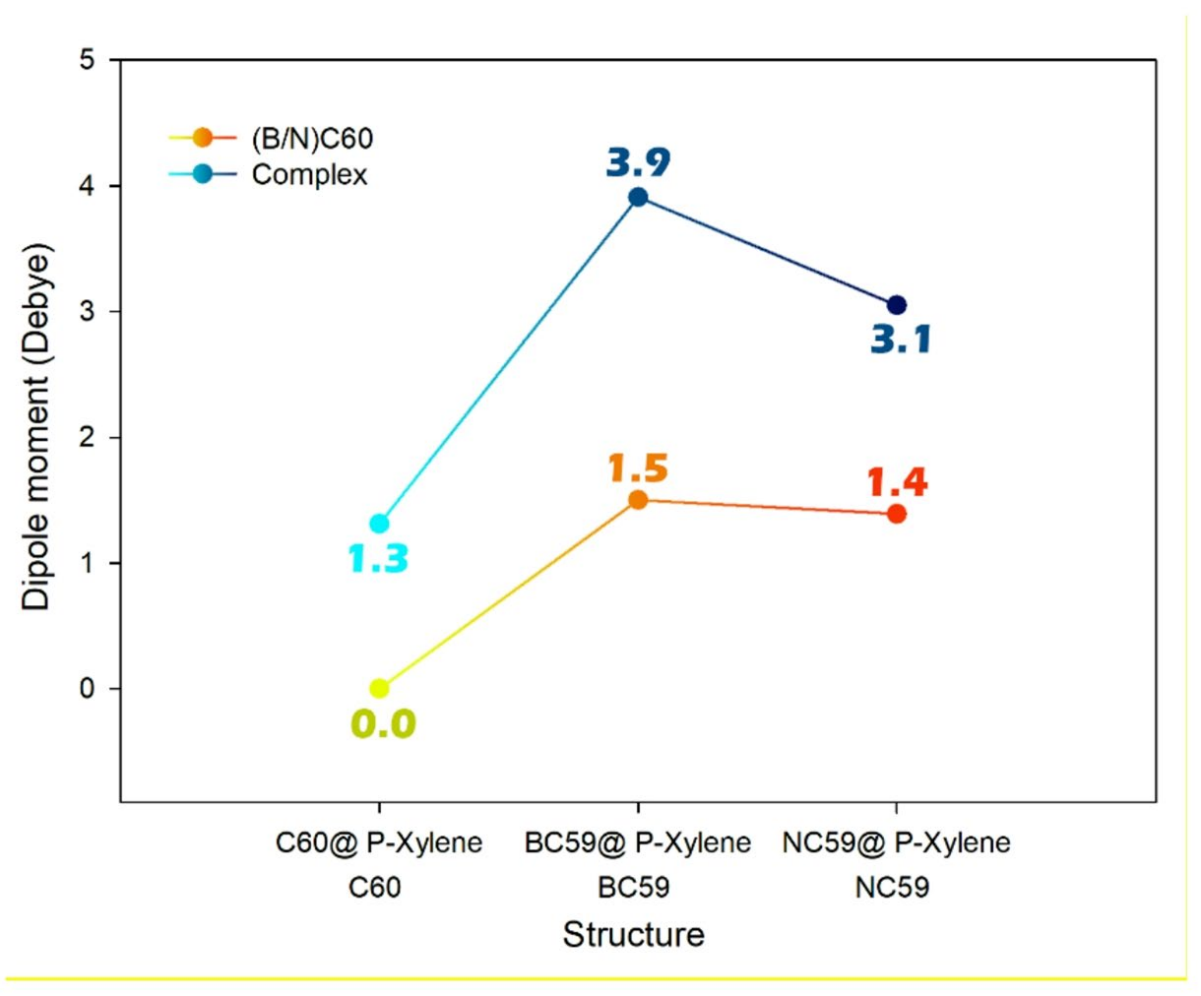
The trend of dipole moment changes in each of the designed structures.

The study of dipole moment is important for sensor design because it gives key information about a molecule’s polarity. This polarity affects both solubility and electrical responsiveness when an analyte is present. A higher dipole moment usually improves solubility in polar solvents like water. This is crucial for using sensors in biological or environmental settings^72–74^. Additionally, changes in the dipole moment upon analyte adsorption indicate electronic rearrangements in the sensor-analyte system. These changes can cause noticeable variations in the electrical signal, which are essential for electrochemical sensing mechanisms^75,76^. In this context, the dipole moment for each designed sensor was computationally studied with and without p-xylene, and the results are shown in Fig. 9.

The dipole-moment variations across C_60_, BC_59_, and NC_59_ in the presence and absence of p-xylene indicate how molecular polarization changes upon adsorption, which in turn correlates with adsorption strength. Pristine C_60_ shows no intrinsic dipole, but acquires a small moment when interacting with p-xylene, reflecting weak polarization and therefore weak adsorption. In contrast, BC_59_ and NC_59_ possess inherent dipoles due to heteroatom substitution, and both exhibit substantial increases in dipole moment upon p-xylene binding. The larger dipole enhancement in BC_59_ (from 1.50 to 3.91 D) compared with NC_59_ (from 1.39 to 3.05 D) suggests a stronger electronic perturbation of the BC_59_ framework and thus a stronger adsorbate (substrate interaction). Since adsorption energies generally become more negative as charge redistribution and polarization intensify, the trend of increasing dipole moment (C_60_ < NC_59_ < BC_59_) matches the trend of adsorption energies, with BC_59_ showing the strongest adsorption, NC_59_ moderate adsorption, and C_60_ the weakest adsorption.

#### Natural bond orbital analysis

Natural Bond Orbital (NBO) analysis is essential for comprehending the intricate electronic structure and interactions within molecular systems, particularly in sensor design^77^. This method elucidates donor-acceptor interactions that occur when an analyte interacts with a sensor, thereby facilitating charge transfer and leading to alterations in the sensor’s electronic properties that generate measurable electrochemical signals^78^. A pivotal aspect of NBO analysis is the second-order perturbation energy matrix (E^2^), which quantifies the stabilization energies resulting from these donor-acceptor interactions. High E^2^ values imply strong interactions between filled (donor) and empty (acceptor) orbitals, which enhance the overall electrical conductivity of the material and amplify the sensor’s response upon analyte binding^79,80^. Within the scope of NBO analysis, several key orbital transitions are examined: σ→σ* transitions involve interactions between bonding and antibonding sigma orbitals; π→π* transitions pertain to transitions between bonding and antibonding pi orbitals; lone pair (LP)→σ* transitions represent the donation of electrons from non-bonding orbitals into antibonding sigma orbitals; and LP→π* transitions involve the transfer from non-bonding orbitals into antibonding pi orbitals^81^. Among these, the π→π* transitions are of particular importance and are often considered as electronic templates because they indicate the presence of delocalized electron systems that are highly sensitive to environmental perturbations, such as the interaction with an analyte^82^. These transitions, by dictating the behavior of electron delocalization and charge distribution, play a crucial role in modulating the electrochemical response, thereby enhancing both the selectivity and sensitivity of the sensor.

**Table 5:** Calculated values ​​of NBOs analysis for the studied complexes.

Complex	Donor (i)	Type	cceptor (j)	Type	E^(2)^ kcal.mol^− 1^	E(j)-E(i) a.u.	F(i, j) a.u.
C_60_@p-xylene	C1-C2	$\:\sigma\:$	C1-C4	$\:{\sigma\:}^{*}$	1.16	1.09	0.045
	C37-C38	$\:\pi\:$	C28-C29	$\:{\pi\:}^{*}$	7.22	0.25	0.054
	C59	LP (1)	C10-C11	$\:{\pi\:}^{*}$	32.40	0.13	0.091
BC_59_@p-xylene	C64-C65	$\:\sigma\:$	B60-C64	$\:{\sigma\:}^{*}$	10.22	1.13	0.136
	C37-C38	$\:\pi\:$	C28-C29	$\:{\pi\:}^{*}$	7.10	0.25	0.054
	C59	LP (1)	C10-C11	$\:{\pi\:}^{*}$	25.52	0.14	0.084
NC_59_@p-xylene	C64-C65	$\:\sigma\:$	C4-N60	$\:{\sigma\:}^{*}$	11.08	0.72	0.117
	C25-C26	$\:\pi\:$	C14-C24	$\:{\pi\:}^{*}$	7.35	0.24	0.053
	C12	LP (1)	C25-C26	$\:{\pi\:}^{*}$	25.37	0.14	0.086

The NBO analysis presented in Table 5 shows the electronic interactions occurring in each complex. In the C_60_@p-xylene complex, three key interactions are observed: a weak σ→σ* interaction (C1-C2 to C1-C4) with an E^(2)^ value of 1.16 kcal/mol, a moderate π→π* interaction (C37-C38 to C28-C29) with an E^(2)^ value of 7.22 kcal/mol, and a strong LP→π* interaction from the lone pair on C_59_ to the π* orbital of C10-C11, with a high stabilization energy of 32.40 kcal/mol, indicating significant electron donation and strong interaction potential. In the BC_59_@p-xylene complex, the σ→σ* interaction (C64-C65 to B60-C64) shows a markedly higher E^(2)^ of 10.22 kcal/mol, suggesting stronger orbital overlap compared to the undoped system. The π→π* interaction is similar in strength (7.10 kcal/mol), while the LP→π* transition shows a slightly lower stabilization energy of 25.52 kcal/mol than in the pristine fullerene, still indicating a substantial electron transfer effect. In the NC_59_@p-xylene complex, the σ→σ* interaction between C64-C65 and C4-N60 has the highest E^(2)^ among all σ interactions at 11.08 kcal/mol, reflecting enhanced orbital interactions due to nitrogen doping. The π→π* transition is also slightly stronger at 7.35 kcal/mol, while the LP→π* interaction from the lone pair on C12 to the π* orbital of C25-C26 registers an E^(2)^ value of 25.37 kcal/mol, nearly identical to that in the boron-doped structure. Overall, the nitrogen- and boron-doped complexes exhibit stronger σ→σ* interactions and comparable π→π* and LP→π* interactions relative to the pristine C_60_, suggesting enhanced electronic communication upon doping. These results support the increased sensing capabilities of BC_59_ and NC_59_ due to their more favorable donor-acceptor interactions, which facilitate efficient charge transfer and contribute to the generation of electrochemical signals upon p-xylene detection.

### NCI analysis

In the field of electrochemical sensors design, Non-Covalent Interaction (NCI) analysis plays a crucial role in understanding the weak intermolecular forces between the sensor surface and the analyte^83^. These interaction forces (e.g., van der Waals forces, hydrogen bonding, π-π stacking) are often responsible for the initial recognition and binding events that enable sensor performance. NCI analysis takes a plot of the Reduced Density Gradient (RDG) versus the product of the sign of the second eigenvalue of the Hessian (λ2), the electron density (ρ), sign(λ_2_).ρ^84^. This plot can effectively apportion the interaction as either attractive, weak, or repulsive: negative sign(λ_2_).ρ = attractive (e.g., hydrogen bonds or π-π interactions); values close to zero = weak, non-directional interactions (e.g., van der Waals); and positive values = repulsive (steric hindrance)^85^. This classification is important in sensor design as non-covalent interactions provide a general approximation for binding affinity, stability in the sensor-analyte complex, and charge transfer efficiency. Therefore, NCI analysis (along with other analyses) will help determine and optimize a sensor property “sensitivity, selectivity, and response time”^86^. As such, NCI and RDG analyses provide essential molecular-level insights that guide the rational design of high-performance electrochemical sensors.

**Fig. 10:**
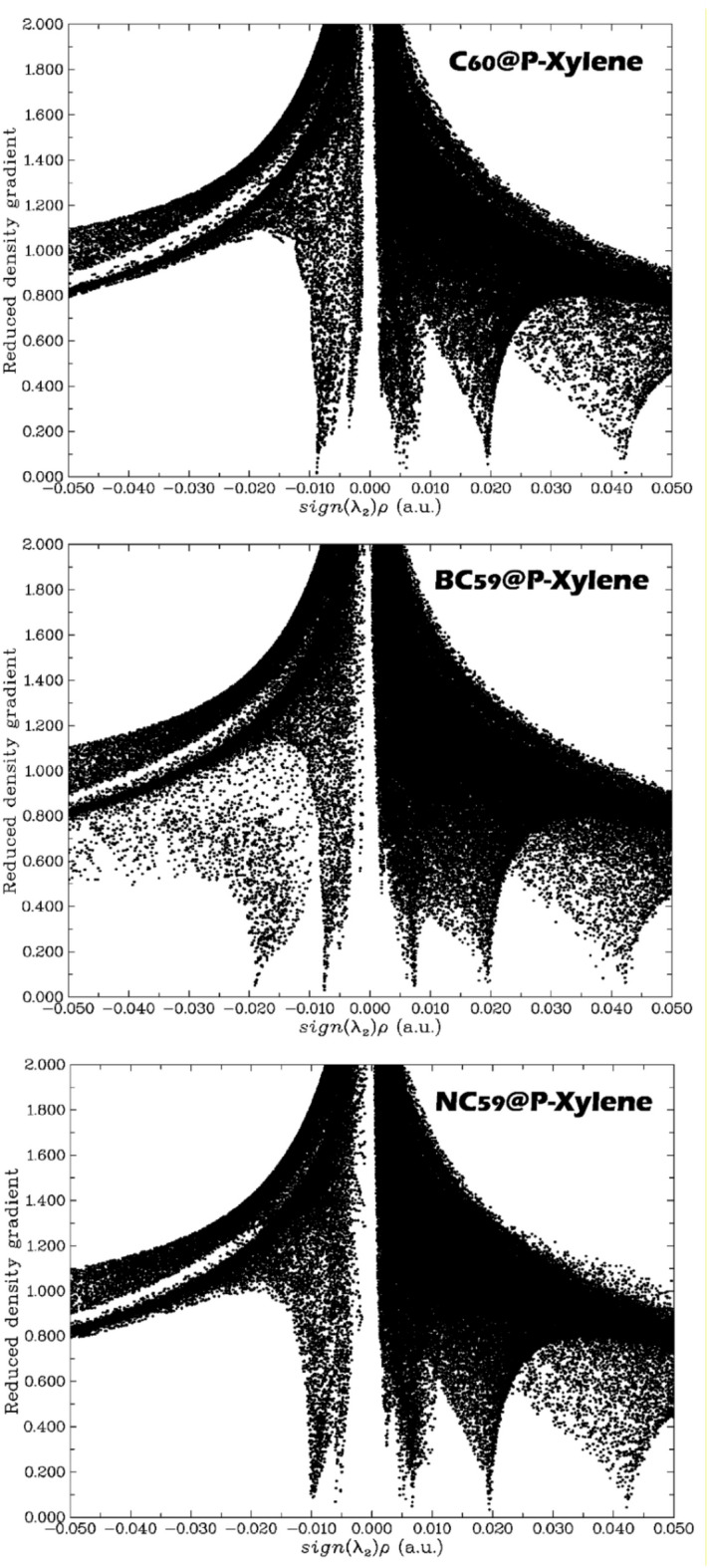
NCI plot for each of the complexes designed in this work.

The NCI plots of the C_60_@p-xylene, BC_59_@p-xylene, and NC_59_@p-xylene complexes reveal important information about the character and strength of the intermolecular forces that enforce sensor-analyte interactions (Fig. 10). In all three complexes, the spike at about zero for sign(λ_2_).ρ denotes the presence of weak van der Waals forces, which are the driving forces for the initial physical adsorption of analyte. The RDG plot of the C_60_@p-xylene complex shows that the interaction is mainly associated with van der Waals forces, as indicated by a narrow distribution centered around zero and only shallow features in the negative region of sign(λ_2_).ρ. Therefore, the complex exhibits a weak interaction consistent with the previously reported moderate adsorption energy and raw recovery time.

In contrast, the BC_59_@p-xylene complex displays a broader and more pronounced distribution extending into the negative region of sign(λ_2_).ρ, signifying stronger attractive interactions such as π-π stacking or possibly B-π interactions due to the presence of the boron dopant. These deeper and more extensive troughs imply enhanced charge transfer and a stronger binding affinity between the sensor and p-xylene, correlating well with the higher adsorption energy, effective charge transport, and desirable sensing characteristics. The NC_59_@p-xylene complex shows interaction features somewhat intermediate between those of C_60_ and BC_59_. The plot includes significant peaks in the negative region, suggesting moderately strong attractive forces likely enhanced by the electronegative nitrogen dopant. This supports the increased interaction strength and stability of the complex, as also reflected in its adsorption and electronic properties.

To give a clearer overview of the nature of non-covalent interactions (van der Waals forces, π-π stacking, and hydrogen bonding) in the adsorption and sensing behavior of each of the designed sensors with/without p-xylene, we summarize here the non-covalent interactions seen in p-xylene each of the fullerene sensors (Table 6).

**Table 6:** Summary of the dominant noncovalent interactions governing p-xylene adsorption on C60, BC59, and NC59, highlighting the roles of Van der Waals forces, – stacking, hydrogen bonding, and – transitions in determining complex stability.

ππππ		
Interaction Type	Description	Observed Interaction in Complex
Van der Waals	Weak, distance-dependent interactions between molecules	Present in all complexes, contributing to the initial adsorption of p-xylene.
π-π Stacking	Interaction between aromatic π-electron systems.	Observed in C_60_@p-xylene and BC_59_@p-xylene, contributing to stronger adsorption in BC_59_.
Hydrogen Bonding	Interaction involving proton donors and acceptors.	Present in NC59@p-xylene, strengthening the complex due to nitrogen’s electron-deficient character
π-π Transitions*	Transfer of electron density between π-orbitals and π* anti-orbitals.	Prominent in BC59 and C60 complexes, especially when the fullerene is doped with boron.

These interactions collectively enhance the affinity of the fullerene sensors for p-xylene and contribute to the observed changes in their electronic properties. For instance, π-π stacking interactions are particularly significant in BC_59_@p-xylene, where the electron-deficient nature of boron dopant promotes stronger π–π interactions with the electron-rich p-xylene. These results are consistent with previous computational findings and confirm the superior performance of BC_59_ as an adsorbent through its enhanced ability to non-covalently interact with the p-xylene analyte. Similarly, van der Waals forces are present across all complexes, supporting initial molecular adsorption. In the NC_59_@p-xylene complex, the presence of hydrogen bonding, facilitated by nitrogen’s electronegativity, plays a key role in stabilizing the complex, further enhancing its electrochemical sensing potential.

### QTAIM

The Quantum Theory of Atoms in Molecules (QTAIM) is a necessary tool for interpreting the character and strength of chemical bonds in molecular complexes, which can be useful in the rational design of electrochemical sensors^87^. The characterization of interactions is conducted by electron density topology and the identification of bond critical points (BCPs). Descriptors such as the Laplacian of the electron density (∇^2^ρ), kinetic energy density (G(r)), potential energy density (V(r)), and the total energy density (Hb = V(r) + G(r)) are used to describe the interaction^88^. These parameters also provide an indication of the extent of covalency or electrostatic character of a bond.

**Fig. 11:**
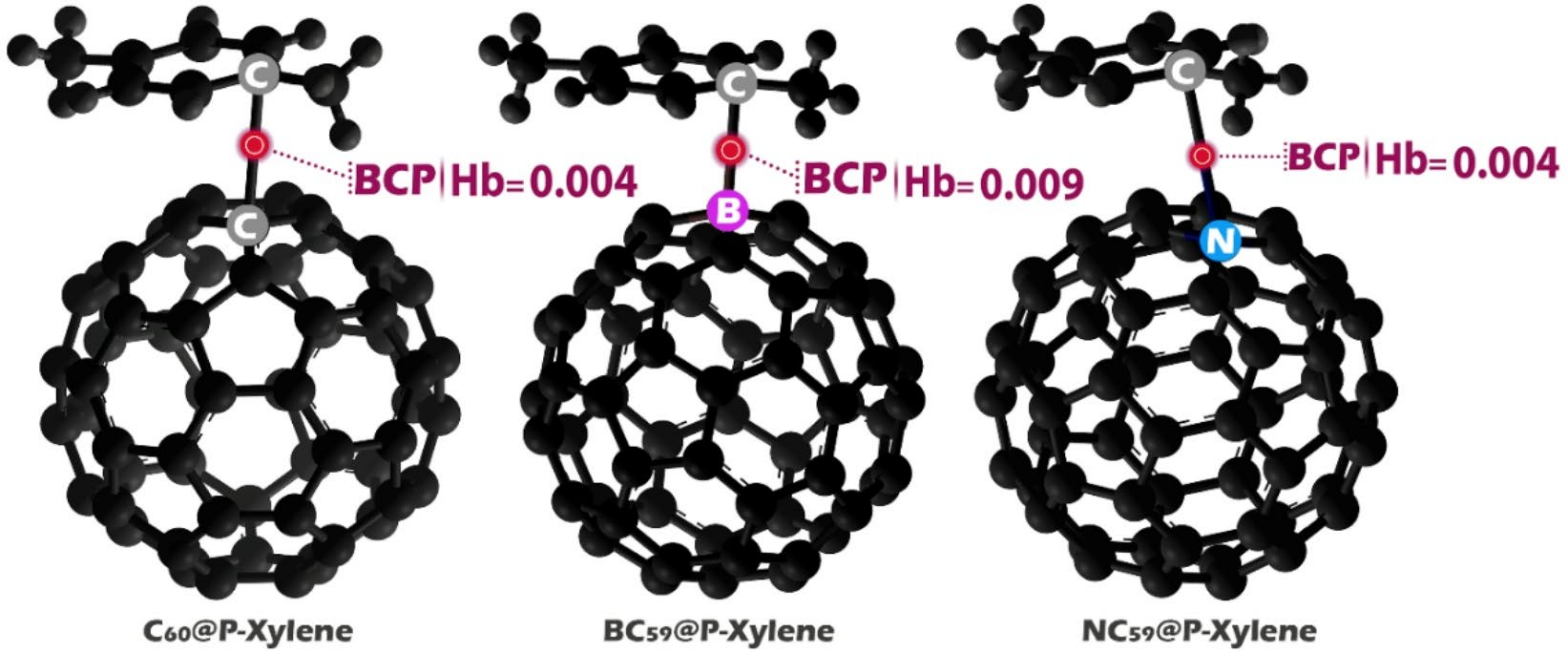
Hb values at the BCP for each of the structures studied in this work.

Per the classification system proposed by Rozas et al., hydrogen bonds can also be classified by the sign and size of ∇^2^ρ and Hb at the BCP^89,90^. Strong hydrogen bonds (Hb < 0, ∇^2^ρ < 0) have a high degree of covalent character and bond strength involving substantial electron sharing. Moderate hydrogen bonds (Hb > 0, ∇^2^ρ < 0) are predominantly electrostatic interactions with intermediate strength and stability. Weak hydrogen bonds (Hb > 0, ∇^2^ρ > 0) are characteristically weak van der Waals based or dispersive interactions with little electron sharing and low binding energy^91,92^. This classification is most useful for determining sensor-analyte interactions. Therefore, the BCP properties were assessed for all designed complexes, and the data are presented in Fig. 11; Table 7.

**Table 7:** QTAIM parameters including electron density ((r)), kinetic energy density (G(r)), potential energy density (V(r)), laplacian of electron density (∇2(r)), and total energy density (Hb) at the bond critical points (BCPs) for the studied sensor-(P-Xylene) complexes.

ρρ				
Structure	ρ(r)	G(r)	V(r)	∇^2^ρ(r)
C_60_@p-xylene	0.008	0.005	−0.001	−0.006
BC_59_@p-xylene	0.019	0.008	0.0007	−0.007
NC_59_@p-xylene	0.010	0.005	−0.001	−0.007

Based on the data, the BC_59_@p-xylene complex has the highest electron density at the bond critical point (ρ(r) = 0.019). It also has the highest kinetic energy density (G(r) = 0.008) and a slightly positive total energy density (Hb = 0.009). While the potential energy density (V(r) = 0.0007) is positive, the Laplacian of the electron density (∇2ρ = −0.007) is negative. The relatively high electron density and the nature of the energy components suggest stable interactions that are mainly electrostatic but have some covalent character. These traits indicate moderate binding strength and stable interactions, which are good for effective adsorption. Therefore, BC_59_ shows the qualities of a strong adsorbent for capturing p-xylene molecules.

Similarly, the C_60_@p-xylene complex exhibits moderate electron density (ρ(r) = 0.008), slightly negative potential energy (V(r) = −0.001), and a small positive total energy density (Hb = 0.004). The negative Laplacian (∇^2^ρ = −0.006) also supports the presence of weak to moderate attractive interactions, likely dominated by van der Waals forces. These parameters suggest that C_60_ also acts as an efficient adsorbent with stable non-covalent interactions conducive to analyte retention.

In contrast, the NC_59_@p-xylene complex shows an intermediate electron density (ρ(r) = 0.010) and energy values similar to those of C_60_, but its slightly higher ρ(r) and equal Hb (0.004) imply an optimized balance between interaction strength and reversibility. This is an important feature in electrochemical sensor applications, where a rapid and reversible interaction is essential for signal generation and recovery. Furthermore, the QTAIM data aligns with previously reported dipole moment and electrical conductivity values for NC_59_, further supporting its suitability as a sensitive and responsive electrochemical sensor for p-xylene detection.

This confirms the dual application potential of these materials: BC_59_ and C_60_ for environmental or industrial filtration of p-xylene, and NC_59_ for real-time, sensor-based detection in medical diagnostics or environmental monitoring.

### Comparison with other literature

Yadav et al. investigated epinephrine adsorption on doped C_60_ fullerenes and reported adsorption energies in the range of −39.5 to −47.8 kJ.mol^− 1^, with the most active doped system showing a HOMO-LUMO gap reduction from 1.62 eV to 1.15 eV upon analyte adsorption^93^. Compared to these results, the BC_59_@p-xylene complex in the present study exhibits a stronger adsorption energy (−55.36 kJ/mol) and a comparable low energy gap (1.21 eV), indicating enhanced adsorption and similar charge transfer improvement.

Okon et al. examined Cu-Ti@C_60_ sensors for toxic gas detection and found adsorption energies between − 42.1 and − 50.3 kJ/mol, with moderate band gap reductions (from ~ 1.68 eV to ~ 1.35 eV). Again, our BC_59_ system outperforms in terms of adsorption energy, while the NC_59_ system achieves a more pronounced band gap narrowing to 0.73 eV, suggesting higher reactivity and sensitivity^94^.

Omidvar and Soleymani studied bimetallic acetone sensors with adsorption energies around − 31 to −41 kJ/mol and final energy gaps above 1.2 eV. These values are significantly less favorable than those obtained in the present work, where both BC_59_ and NC_59_ complexes display higher adsorption strength and, in the case of NC_59_, a much lower energy gap, indicating superior charge transport capability^95^.

Ogunwale et al. reported that Ir- and Au-decorated C_60_ sensors for drug detection had adsorption energies in the range of −34.5 to −46.2 kJ/mol, with the lowest energy gaps around 1.26 eV after adsorption. Compared to this, the BC_59_ system achieves both stronger adsorption and similar or better band gap reduction, while NC_59_ offers a far greater decrease in the gap (0.73 eV), highlighting the remarkable sensitivity of the present design^96^.

Overall, relative to these previous studies, the doped C_60_ systems designed here (especially BC_59_ for adsorption and NC_59_ for electrochemical sensing) demonstrate superior adsorption strength, greater modulation of the HOMO-LUMO gap, and enhanced electrical conductivity. These improvements directly translate to higher sensitivity and more efficient signal generation, positioning the proposed sensors as advanced alternatives for both biomedical and environmental detection of p-xylene.

### Future work

Recent work in nanosensor development shows a steady movement toward devices with enhanced sensitivity, portability, and performance driven by advanced materials. Graphene-platinum paper-based microfluidic biosensors demonstrate how combining conductive nanomaterials with foldable, low-cost substrates enables rapid, smartphone-assisted detection of biological markers^97^. Also, reviews of MXenes highlight their high surface area, tunable chemistry, and strong charge transport, which make them suitable for applications ranging from biosensing to environmental remediation^98^. These studies reveal that continual improvements in materials engineering and fabrication methods are expanding the versatility and precision of modern sensing technologies.

Within this broader context, the current findings suggest that BC_59_ and NC_59_ nanostructures offer promising potential for p-xylene detection and warrant experimental synthesis and characterization using TEM, XPS, and Raman spectroscopy to verify their predicted properties. Incorporating these doped fullerenes into sensor configurations such as thin-film electrodes, field-effect transistors, or colorimetric devices would enable empirical testing of their performance. BC_59_ may serve as an efficient adsorbent for environmental cleanup, while NC_59_ could function as the core sensing material in compact, fast-responding electrochemical diagnostic systems. Advancing these materials from theoretical modeling to real-world platforms would support improved disease-related monitoring, pollution tracking, and early-warning surveillance, reinforcing the broader societal value of this research.

## Conclusion

In this study, a comprehensive analysis was performed to evaluate the potential of pristine C_60_ fullerene and its boron (BC_59_) and nitrogen (NC_59_) doped derivatives for the detection of p-xylene. Various computational methods, including density functional theory (DFT) and Quantum Theory of Atoms in Molecules (QTAIM), were employed to investigate the electronic properties, adsorption behavior, and sensor capabilities of these materials.

The bond lengths and angles of the fullerene structures were evaluated to understand how doping with boron and nitrogen influences the geometry of the material. For pristine C_60_, the bond lengths ranged from 1.40 to 1.45 Å for C-C bonds, while the bond angles were approximately 108° to 120°. After boron doping (BC_59_), the C-B bond lengths increased to 1.53–1.55 Å, indicating a weakening of the bonds and a localized geometric distortion due to boron’s electron deficiency. In contrast, nitrogen doping (NC_59_) resulted in shorter C-N bond lengths (1.40–1.42 Å) and more stable bond angles (close to the values of C_60_), reflecting nitrogen’s higher electronegativity and its ability to attract π-electrons more effectively than boron. Cohesive energy calculations, which assess the stability of the material, showed that doping slightly reduced the structural stability of the pristine C_60_. The cohesive energy of C_60_ was − 197.3 kcal/mol, and upon doping, it decreased to −195.9 kcal/mol for BC59 and − 196.1 kcal/mol for NC_59_. The reduction in cohesive energy suggests a minor destabilization due to doping, with nitrogen doping (NC_59_) proving to be slightly more stable than boron doping (BC_59_), though the difference was not significant enough to affect their thermodynamic stability.

The energy gap (HLG) between the highest occupied molecular orbital (HOMO) and the lowest unoccupied molecular orbital (LUMO) is an important indicator of reactivity and sensor performance. The energy gap for pristine C_60_ was measured at 1.68 eV, which means it has moderate reactivity. After doping, the energy gap for BC_59_ dropped to 1.35 eV and for NC_59_ to 1.41 eV, showing increased reactivity due to the addition of heteroatoms. When interacting with p-xylene, the energy gaps decreased even further. NC_59_ experienced the largest drop to 0.73 eV, indicating a significant boost in reactivity, which benefits sensor applications. This reduction in the energy gap points to better charge transfer abilities and improved sensor performance. The maximum charge transfer (ΔNmax) values were calculated to assess the charge donation or acceptance capacity of each system. Pristine C60 had a ΔNmax of 5.40. BC59 rose to 6.30, and NC_59_ reached the highest value of 6.37. This shows that doping with boron and nitrogen improves the fullerene’s ability to accept or donate charge, with nitrogen doping (NC_59_) offering the best charge transfer capacity. Regarding electrical conductivity (σ), NC_59_ showed a significant increase (3.68 × 10^− 3^ to 2.07 × 10^3^), highlighting its high potential for electrochemical sensing applications.

The recovery time (τ), which indicates how quickly the sensor returns to its original state after detecting the analyte, was also calculated. NC_59_ exhibited the fastest recovery time of 3.5 × 10^− 7^ s, followed by C_60_ with 9.8 × 10^− 7^ s, and BC_59_ with a slower recovery time of 1.9 × 10^− 5^ s. This rapid recovery time of NC_59_ makes it particularly suitable for real-time detection applications, where quick responses are essential, such as in biomedical diagnostics for early cancer detection. Adsorption energies (Eads) were evaluated to determine the strength of the interaction between p-xylene and each sensor. BC_59_ showed the strongest interaction with p-xylene, with an adsorption energy of −41.68 kJ/mol, followed by C_60_ at −34.30 kJ/mol, and NC_59_ at −31.71 kJ/mol. This indicates that while BC_59_ is the best candidate for environmental applications requiring strong adsorption, NC_59_, despite weaker binding, exhibits exceptional conductivity and fast recovery, making it more suitable for electrochemical sensing.

The dipole moment was analyzed to understand the polarity of the materials and their interaction with p-xylene. C_60_ showed no intrinsic dipole moment but acquired a small moment upon adsorption of p-xylene. In contrast, BC_59_ and NC_59_ had inherent dipoles due to the heteroatom substitution, with BC59 showing a larger increase in dipole moment (from 1.50 to 3.91 D) compared to NC_59_ (from 1.39 to 3.05 D). This suggests that BC_59_ experiences a stronger electronic perturbation upon adsorption, which correlates with its stronger binding and higher adsorption energy. NBO analysis revealed that interactions between p-xylene and the fullerene-based sensors occurred through donor-acceptor interactions, with the lone pairs of electrons on the fullerene interacting with the π* orbitals of p-xylene. BC_59_@p-xylene exhibited the strongest donor-acceptor interactions, followed by NC_59_@p-xylene and C60@p-xylene. The stabilization energies from these interactions (E^2^ values) were highest for BC_59_, indicating that boron doping enhances the strength of the interactions, which is consistent with the higher adsorption energy and better sensing performance observed for BC_59_.

NCI analysis provided insights into the nature of the intermolecular forces at play in the sensor-analyte interactions. In all three complexes, van der Waals forces played a significant role in the initial adsorption of p-xylene. BC_59_@p-xylene displayed stronger attractive forces, likely due to π-π stacking interactions enhanced by the boron dopant, while NC_59_@p-xylene exhibited moderate interactions, with the presence of hydrogen bonding contributing to its adsorption strength. QTAIM analysis further characterized the nature of the chemical bonds in the complexes. BC_59_@p-xylene showed the highest electron density at the bond critical point (ρ(r) = 0.019), indicating stable interactions. The kinetic energy density (G(r) = 0.008) and total energy density (Hb = 0.009) also supported the stability of the BC_59_@p-xylene complex, which was characterized by moderate binding strength and favorable interaction stability. The computational analyses revealed that BC_59_ and NC_59_ doped fullerenes exhibit superior properties for the detection of p-xylene compared to pristine C_60_. BC_59_ offers the best adsorption strength and is suitable for environmental applications, while NC_59_ provides the highest reactivity, fast recovery, and exceptional conductivity, making it ideal for electrochemical sensing, particularly in medical diagnostics. These findings suggest that both BC_59_ and NC_59_ could play significant roles in advancing sensing technologies for environmental monitoring and early cancer detection, with NC_59_ being particularly promising for real-time applications.

In addition to the promising theoretical results, the experimental feasibility of synthesizing BC_59_ and NC_59_ involves doping C_60_ fullerenes with boron or nitrogen atoms using methods such as chemical vapor deposition (CVD) or arc discharge, followed by electrode fabrication through techniques like screen-printing or sputtering to create thin-film sensors. These sensors can be further tested for their real-time p-xylene detection capabilities in environmental or biomedical settings. It is important to note that the current computational models did not account for temperature dependence, solvent dynamics, or the explicit orientation of p-xylene, which could affect adsorption energies and sensor performance. Future studies incorporating these factors will enhance the accuracy of the predictions and provide a more comprehensive understanding of the sensor behavior in practical applications.

## Data Availability

All data generated or analyzed during this study are included in this published article.
